# An Observational Study: Investigating White Matter Tract Alterations in Females Across Age Groups Using Diffusion Tensor Imaging

**DOI:** 10.7759/cureus.76975

**Published:** 2025-01-05

**Authors:** Bhamini Sharma, Anjana Mittal, Amit Mittal, Rahul Sharma, Harpreet Kaur Mohel, Rashmi Malhotra, Kirandeep K Aulakh

**Affiliations:** 1 Anatomy, Maharishi Markandeshwar (Deemed to be University), Mullana, IND; 2 Radiodiagnosis, Maharishi Markandeshwar (Deemed to be University), Mullana, IND; 3 Anatomy, All India Institute of Medical Sciences, Rishikesh, Rishikesh, IND

**Keywords:** brain mri normal, • diffusion tensor imaging (dti), • fractional anisotropy (fa), mean diffusivity (md), white matter changes on mri

## Abstract

Introduction

White matter tracts in the brain are essential for neurocognitive processes. Understanding their integrity is crucial for assessing both healthy and pathological brain states. Tractography based on diffusion tensor imaging (DTI) provides a non-invasive method to map and measure these pathways. The purpose of this study is to examine the 10 main white matter tracts in females aged 18 to 50, focusing on mean diffusivity (MD), volume, and fractional anisotropy (FA).

Materials and methods

Fifty-one healthy females aged 18-50 years participated in the study, divided into three age groups: 18-30, 31-40, and 41-50 years. DTI scans were acquired using a Philips Multiva 1.5T MRI machine. The analyzed white matter tracts included the Inferior Fronto-occipital Fasciculus (IFOF), Superior Fronto-occipital Fasciculus (SFOF), Inferior Longitudinal Fasciculus (ILF), Superior Longitudinal Fasciculus (SLF), Cingulum, Corticospinal Tract (CST), Forceps Major, Forceps Minor, Uncinate Fasciculus, and Anterior Thalamic Radiation (ATR). Volume, FA, and MD were measured and statistically analyzed using the Kruskal-Wallis Test.

Results

The analysis revealed no significant differences in most parameters across age groups. However, significant increases in MD for the SLF and Cingulum in the older age groups suggested potential age-related changes in these pathways. Despite these findings, many other tract parameters did not show statistically significant differences across age groups.

Discussion

The study highlights potential age-related deterioration in white matter, especially in the SLF and Cingulum, as indicated by higher MD in older age groups. This suggests that white matter may play a role in neurodegenerative processes and cognitive decline. The ability to identify subtle white matter alterations before clinical symptom onset underscores the value of DTI-based tractography in early identification of neurological disorders such as schizophrenia and Alzheimer's disease.

Conclusion

This study provides valuable insights into the age-related changes in white matter integrity in females aged 18-50, particularly with respect to MD increases in the SLF and Cingulum. The findings underscore the importance of DTI in clinical diagnostics and the early detection of neurodegenerative conditions. Further research with larger, more diverse samples is necessary to explore the influence of additional factors such as hormonal changes and lifestyle on white matter integrity across the lifespan.

## Introduction

The brain functions as a complex, interconnected organ, engaging in sophisticated and dynamic neurocognitive activities [1]. To effectively study the brain's cortex and its functional areas, anatomical advancements are essential. Since each individual possesses a distinct network of connections among various brain regions, a comprehensive examination is necessary to comprehend the brain's functioning [2].

Utilizing tractography allows for the visualization of white matter pathways and the quantitative analysis of specific tracts [3]. The field of diagnostics has been revolutionized by magnetic resonance imaging, or nuclear magnetic resonance imaging (NMR) [4]. The discovery of NMR by Bloch and Purcell in 1946, for which they received the Nobel Prize in Physics in 1952, marked the beginning of rapid advancements in this technology [5].

Diffusion tensor imaging (DTI)-based three-dimensional tractography is emerging as a key technique for exploring human white matter architecture [6]. This approach reconstructs white matter fibers by estimating the local orientation of nerve fibers voxel by voxel [7]. It enables the visualization of specific white matter bundles and potentially quantifies the characteristics of individual tracts, allowing for the evaluation of how illnesses impact white matter integrity [8]. Quantitative analysis methods in diffusion tensor imaging encompass region of interest-based approaches, voxel-based analysis, and tractography [9].

The present study aims to characterize ten major white matter tracts in females, focusing on volume, fractional anisotropy (FA), and mean diffusivity (MD) derived from DTI scans. By analyzing these parameters, we aim to enhance our understanding of how age and other factors influence white matter integrity in this demographic.

## Materials and methods

Study design

This descriptive observational study was conducted at the Department of Anatomy, MMIMSR, Mullana, Ambala, Haryana, India. It was approved by the Research Ethics Committee under Project No. IEC - 2206.

Inclusion and exclusion criteria

The study included 51 females aged 18-50 years. Only those subjects who were willing to participate and had no brain injury, head trauma, signs of neurological disorders, confusion, or any past history of brain-related injury were included in the study. Prior written and verbal consent for the study was obtained from all the subjects, both in English and in the vernacular. Medical records of the participants were reviewed for any neurological disorder, brain tumor, or brain injury, which led to the exclusion of 49 participants from the study.

Data collection

Data was collected using a Philips Multiva 1.5T MRI machine in the Department of Radiology, MMIMSR, Mullana, Ambala, Haryana, India. DTI scans were taken of the subjects with prior written and verbal consent. The tracts included in the study were the inferior fronto-occipital fasciculus (IFOF), superior fronto-occipital fasciculus (SFOF), inferior longitudinal fasciculus (ILF), superior longitudinal fasciculus (SLF), cingulum, corticospinal tract (CST), forceps major, forceps minor, uncinate fasciculus, and anterior thalamic radiation (ATR). The volume of each tract was estimated, providing a measure of the spatial extent of the tracts within the brain. FA and MD, which provide information about fiber density, axonal diameter, and nerve cell orientation, were obtained.

Statistical analysis

In this study, three age groups were created: Group 1 (18-30 years), Group 2 (31-40 years), and Group 3 (41-50 years). The data were entered into an Excel sheet, with a double-entry process implemented to minimize human error. IBM SPSS Statistics for Windows, Version 28.0 (Released 2021; IBM Corp., Armonk, New York, United States) was used for statistical analysis. The data were analyzed using the Shapiro-Wilk test and were not normally distributed across these age categories, so non-parametric tests (Kruskal-Wallis test) were used for group comparisons. The p-value considered statistically significant was < 0.05 in our study.

## Results

In our study, 51 females aged between 18 and 50 years were included. The sample was divided into three age groups: Group 1 (18-30 years, n=15), Group 2 (31-40 years, n=7), and Group 3 (41-50 years, n=27). Table 1 presents the mean and standard deviation for the volume, FA, and MD of the ten white matter tracts examined. It summarizes the relationship between age and the structural parameters (volume, FA, MD) of several brain tracts in females aged 18-50 years. The analysis included three age groups: 18-30 years (n=15), 31-40 years (n=7), and 41-50 years (n=27). The Kruskal-Wallis and Chi-square tests were used to assess the significance of differences across the age groups. While some tracts showed trends towards differences in MD and FA, most parameters across different age groups did not reach statistical significance. Notable findings include significant increases in MD for older age groups in the SLF and Cingulum, suggesting potential age-related changes in these specific pathways. Figures below depict the DTI images of the various tracts in different colors. The scales in the images represent the anterior and posterior sides of the scan, showing the direction of the fibers of the tracts in brain tractography.

**Table 1:** Association between age and volume, FA, and MD examined in females aged 18-50 years. *Significant at p<0.05, Kruskal-Wallis test, chi-square test (χ2) Voxel size (spatial resolution): 2.5×2.5×2.5 mm³ (isotropic); b-value(s): 1000 s/mm²; motion correction and eddy current distortion correction were performed using FSL's eddy tool. Brain extraction was performed using FSL's BET tool. IFOF: Inferior Fronto-occipital Fasciculus; SFOF: Superior Fronto-occipital Fasciculus; ILF: Inferior Longitudinal Fasciculus; SLF: Superior Longitudinal Fasciculus; CST: Corticospinal Tract; ATR: Anterior Thalamic Radiation; FA: Fractional Anisotropy; MD: Mean Diffusivity; mm3: Cubic Millimeters; s: Seconds; mm2 s−1: Square Millimeters per Second.

Tracts	Parameters - Volume (mm^3^ ), MD (×10^−3^ mm^2^ s^ −1^ )	Age			P-value	Chi-square (χ2)
		18-30 years (n = 15)	31-40 years (n = 7)	41-50 years (n = 27)		
IFOF	Volume	10.71 ± 5.06	10.91 ± 7.20	10.16 ± 5.03	0.927	0.153
	FA	0.48 ± 0.03	0.50 ± 0.04	0.48 ± 0.04	0.511	1.344
	MD	0.89 ± 0.04	0.89 ± 0.06	0.98 ± 0.22	0.571	1.122
SFOF	Volume	11.05 ± 5.99	10.12 ± 7.44	10.44 ± 5.68	0.610	0.988
	FA	0.50 ± 0.04	0.50 ± 0.04	0.48 ± 0.04	0.421	1.732
	MD	0.86 ± 0.06	0.85 ± 0.08	0.98 ± 0.19	0.088	4.864
SLF	Volume	8.67 ± 4.39	9.14 ± 5.44	10.88 ± 3.26	0.106	4.487
	FA	0.49 ± 0.05	0.49 ± 0.04	0.45 ± 0.05	0.060	5.627
	MD	0.92 ± 0.06	0.88 ± 0.09	1.00 ± 0.15	0.031***	6.93
ILF	Volume	9.83 ± 6.34	7.79 ± 5.42	9.46 ± 5.11	0.372	1.976
	FA	0.49 ± 0.05	0.49 ± 0.04	0.48 ± 0.05	0.905	0.199
	MD	0.94 ± 0.12	0.89 ± 0.07	0.94 ± 0.10	0.187	3.356
Cingulum	Volume	10.45 ± 7.63	8.81 ± 7.15	10.63 ± 7.37	0.675	0.786
	FA	0.48 ± 0.04	0.48 ± 0.05	0.49 ± 0.04	0.784	0.488
	MD	0.92 ± 0.24	0.94 ± 0.23	1.03 ± 0.15	0.023***	7.543
CST	Volume	4.19 ± 2.45	8.26 ± 7.66	5.99 ± 2.42	0.079	5.083
	FA	0.52 ± 0.04	0.53 ± 0.04	0.52 ± 0.05	0.752	0.57
	MD	0.86 ± 0.08	0.86 ± 0.10	0.90 ± 0.14	0.937	0.131
Forceps Major	Volume	3.58 ± 2.88	3.42 ± 4.36	4.02 ± 2.89	0.650	0.862
	FA	0.49 ± 0.06	0.48 ± 0.05	0.48 ± 0.06	0.948	0.108
	MD	0.88 ± 0.07	0.93 ± 0.15	0.98 ± 0.13	0.102	4.564
Forceps Minor	Volume	2.36 ± 1.26	3.21 ± 2.22	4.01 ± 2.95	0.205	3.167
	FA	0.50 ± 0.04	0.49 ± 0.06	0.46 ± 0.06	0.198	3.241
	MD	0.90 ± 0.08	0.86 ± 0.21	0.88 ± 0.10	0.763	0.541
Uncinate Fasciculus	Volume	2.00 ± 1.27	2.51 ± 2.09	3.25 ± 1.58	0.089	4.842
	FA	0.47 ± 0.03	0.47 ± 0.06	0.43 ± 0.06	0.212	3.105
	MD	0.88 ± 0.08	0.89 ± 0.09	0.93 ± 0.12	0.389	1.889
ATR	Volume	1.90 ± 1.49	1.84 ± 1.30	2.31 ± 1.33	0.411	1.78
	FA	0.48 ± 0.04	0.48 ± 0.05	0.47 ± 0.03	0.748	0.58
	MD	0.87 ± 0.06	0.87 ± 0.07	0.85 ± 0.11	0.991	0.017

**Figure 1:**
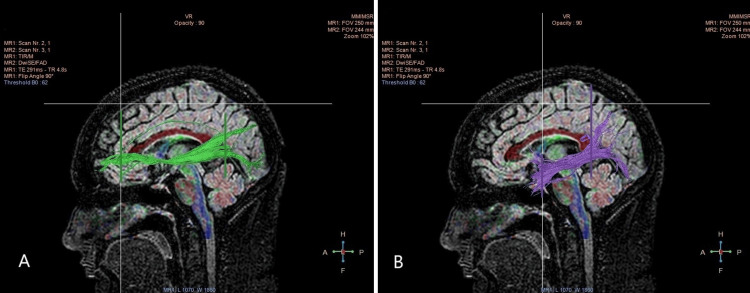
DTI scan images of the tracts, A: IFOF depicted in green, B: ILF depicted in purple. IFOF: Inferior fronto-occipital fasciculus; ILF: Inferior longitudinal fasciculus; DTI: Diffusion tensor imaging.

Figure 1 shows the DTI scan images of two important white matter tracts in the brain: the Inferior Front-Occipital Fasciculus (IFOF) and the ILF. The IFOF, shown in green, is a key white matter network that links the frontal and occipital lobes. The ILF, represented in purple, is a lengthy bundle of fibers that runs along the lateral aspect of the temporal lobe. Both tracts are essential for various sensory and cognitive functions, and their study through DTI helps us understand how structural integrity might relate to age-related changes in the brain.

**Figure 2:**
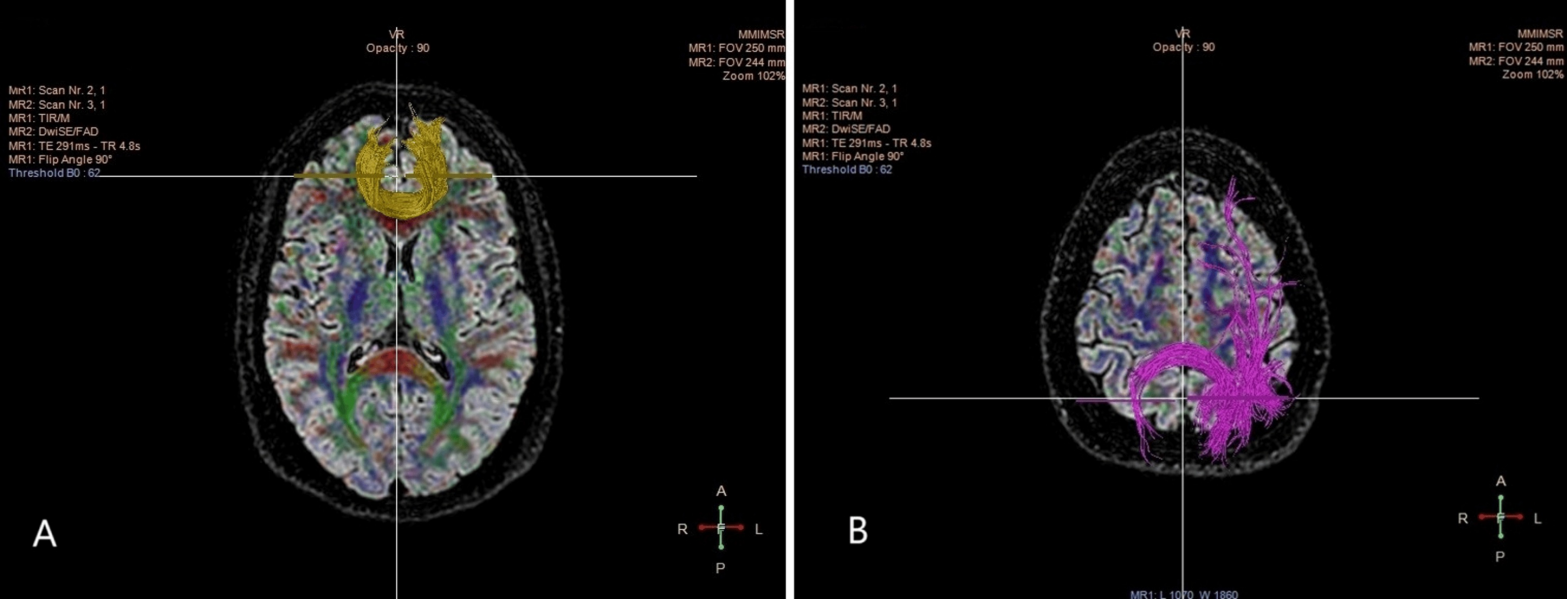
DTI scan images of the tracts: A: Forceps minor depicted in yellow; B: Forceps major depicted in pink. DTI: Diffusion tensor imaging.

The DTI scan images of the Forceps Major and Forceps Minor, two sections of the Forceps tract, are shown in Figure 2. The pink-colored Forceps Major is a sizable bundle of fibers that connects the brain's left and right hemispheres, especially the occipital lobes. The yellow-colored Forceps Minor is a smaller bundle of fibers that links the frontal lobes in both hemispheres. These two tracts are important for interhemispheric communication, and examining their structural changes with age may help us understand age-related differences in cognitive and motor functions.

**Figure 3:**
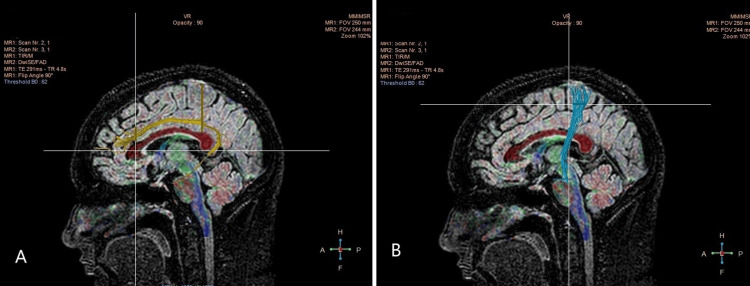
DTI scan images of the tracts, A: ATR depicted in blue, B: Uncinate Fasciculus depicted in orange. DTI: Diffusion tensor imaging; ATR: Anterior Thalamic Radiation.

Figure 3 presents the Cingulum and Corticospinal Tract (CST), two crucial white matter tracts involved in various brain functions. The yellow-highlighted Cingulum is a notable bundle of fibers that surrounds the corpus callosum and connects the frontal, parietal, and temporal lobes, among other areas of the brain. The CST, seen in blue, is a lengthy conduit that runs from the motor cortex to the spinal cord. Examining these tracts with DTI helps to understand how changes in these pathways occur as people age.

**Figure 4:**
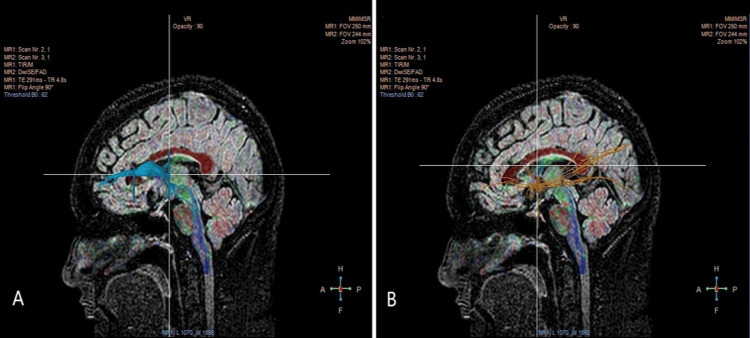
DTI scan images of the tracts: A: ATR depicted in blue; B: Uncinate Fasciculus depicted in orange. DTI: Diffusion tensor imaging; ATR: Anterior Thalamic Radiation.

Figure 4 showcases the Anterior Thalamic Radiations (ATR) and the Uncinate Fasciculus, two important tracts for various brain functions. The ATR, depicted in blue, is a bundle of fibers that links the thalamus and prefrontal cortex. The orange-depicted Uncinate Fasciculus connects the frontal and temporal lobes.

## Discussion

Ten major white matter tracts in females aged 18 to 50 years were characterized utilizing DTI to observe changes in white matter integrity as influenced by age. The findings reveal important insights into the structural properties of these tracts, specifically regarding volume, FA, and MD. Our results demonstrated significant increases in MD for the SLF (p<0.031) and the Cingulum (p<0.023) in older age groups, indicating potential degradation or alterations in these pathways associated with aging. Decreases in FA and increases in MD with age suggest a general deterioration in white matter integrity in healthy aging, which might be caused by underlying myelin alterations as suggested by Bennett IJ and Madden DJ (2010) [10]. Deterioration of frontocerebellar neuronal nodes and connecting circuitry may play a major role in the prominent, persistent, and crippling motor deficits of alcoholism, including ataxia and visuospatial impairment, as reported by Sullivan EV and Pfefferbaum A (2005) [11].

According to Taki Y et al. (2011), certain tracts, such as the SLF, tend to maintain their structural integrity better over time [12]. Longitudinal research by Resnick SM et al. (2003) showed that white matter integrity significantly decreased over time, particularly in elderly individuals [13]. Hasan KM et al. (2009) stated that the uncinate fasciculus has been utilized as a marker for growth, natural aging, and surgically-induced separation of the frontal and temporal lobes [14].

Quantifying changes in white matter tracts can offer valuable insights into neurological and psychiatric disorders that exhibit age and gender-related differences. For instance, Alzheimer's and dementia are typically associated with compromised white matter integrity, especially in the IFOF and Cingulum, highlighting that the anatomical organization of networks within the human brain is linked to sex and brain size. Understanding the baseline values of these tracts in healthy individuals is essential for establishing a reference point to detect early pathological changes in clinical settings [15-17]. Knowing the white matter's spatial arrangement facilitates finding the safest corridor, especially in deeply located lesions [18,19]. Deterioration of frontocerebellar neuronal nodes and connecting circuitry may also play a major role in the prominent, persistent, and crippling cognitive and motor deficits of alcoholism, including ataxia, executive dysfunction, and visuospatial impairment, according to MRI studies [20].

Limitations

While the study successfully analyzed a representative sample of females, the relatively small size of the age groups, particularly the 31-40 years cohort, limits the generalizability of the findings. Furthermore, the limited population and gender specificity restrict the study. In the future, clinical cases related to neurological disorders can also be included in the study for better understanding and clinical correlation. Investigating the impact of other variables such as hormonal changes or lifestyle factors on white matter integrity can provide a more comprehensive understanding of these pathways across the lifespan.

## Conclusions

Our study provides important new insights into how white matter integrity varies with aging in females between the ages of 18 and 50. The results highlight significant changes, particularly the increases in MD for the SLF and cingulum in older age groups, which may indicate aging-related deterioration of these pathways. The study also underscores the utility of DTI in clinical diagnostics, highlighting its potential as an early detection tool for neurological diseases. The ability to detect small white matter alterations before clinical symptoms appear could lead to targeted therapies and improved diagnostic accuracy. However, the limited sample size and lack of demographic diversity suggest the need for further research. Future studies should focus on larger, more diverse sample sizes and explore other factors affecting white matter integrity, such as lifestyle choices and hormonal fluctuations. A deeper understanding of these connections can help us more accurately assess the implications for cognitive health across the lifespan and develop more effective early intervention strategies for aging populations. Together, these findings contribute to the expanding body of research on brain aging and open the door for further investigations into the anatomical changes underlying cognitive decline.
